# Mitigating a COVID-19 Outbreak Among Major League Baseball Players — United States, 2020

**DOI:** 10.15585/mmwr.mm6942a4

**Published:** 2020-10-23

**Authors:** Meghan T. Murray, Margaret A. Riggs, David M. Engelthaler, Caroline Johnson, Sharon Watkins, Allison Longenberger, David M. Brett-Major, John Lowe, M. Jana Broadhurst, Chandresh N. Ladva, Julie M. Villanueva, Adam MacNeil, Shoukat Qari, Hannah L. Kirking, Michael Cherry, Ali S. Khan

**Affiliations:** ^1^Epidemic Intelligence Service, CDC; ^2^CDC COVID-19 Response Team; ^3^Translational Genomics Research Institute, Phoenix, Arizona; ^4^Philadelphia Department of Health, Pennsylvania; ^5^Pennsylvania Department of Health; ^6^University of Nebraska Medical Center, Omaha, Nebraska.

Mass gatherings have been implicated in higher rates of transmission of SARS-CoV-2, the virus that causes coronavirus disease 2019 (COVID-19), and many sporting events have been restricted or canceled to limit disease spread (*1*). Based on current CDC COVID-19 mitigation recommendations related to events and gatherings (*2*), Major League Baseball (MLB) developed new health and safety protocols before the July 24 start of the 2020 season. In addition, MLB made the decision that games would be played without spectators. Before a three-game series between teams A and B, the Philadelphia Department of Public Health was notified of a team A player with laboratory-confirmed COVID-19; the player was isolated as recommended (*2*). During the series and the week after, laboratory-confirmed COVID-19 was diagnosed among 19 additional team A players and staff members and one team B staff member. Throughout their potentially infectious periods, some asymptomatic team A players and coaches, who subsequently received positive SARS-CoV-2 test results, engaged in on-field play with teams B and C. No on-field team B or team C players or staff members subsequently received a clinical diagnosis of COVID-19. Certain MLB health and safety protocols, which include frequent diagnostic testing for rapid case identification, isolation of persons with positive test results, quarantine for close contacts, mask wearing, and social distancing, might have limited COVID-19 transmission between teams.

## Investigation and Results

On June 23, before the July 24 start of the 2020 season, MLB implemented health and safety protocols, which included COVID-19 mitigation strategies (Table). The health and safety protocols established tiered, risk-based testing for MLB teams, which called for persons who received a positive SARS-CoV-2 test result to be placed in isolation and for close contacts to be quarantined separately. Tier 1 included players and persons with high interpersonal contacts with players (e.g., coaches, umpires, and medical staff members). Tier 2 included persons who were able to wear face masks, maintain social distance, or both during their regular interactions with tier 1 persons (e.g., travel and home clubhouse staff members). Tier 3 included persons with minimal contact with other staff members (e.g., cleaning service and stadium security staff members).

**TABLE:** Selected mitigation strategies implemented by Major League Baseball at the opening of the 2020 season — United States, 2020

Mitigation strategy	Description
Minimize contact between players and staff members (tiers)	To manage within team contacts, relevant players and staff members were divided into three tiers to minimize contact needed on-site for games:
	• Tier 1: players and persons with high interpersonal contacts with players, e.g., coaches and medical staff members
	• Tier 2: nonplaying staff members who work with tier 1 but are able to wear face masks, maintain social distancing recommendations, or both, e.g., traveling and home clubhouse staff members
	• Tier 3: essential staff members who do not require close contact with tier 1, e.g., cleaning service providers and stadium security personnel
	Umpires were not permitted to visit either team’s clubhouse and were limited to umpire room, field, and areas necessary for travel between
	Opposing team players and staff members were not permitted to visit each other’s clubhouse facilities
	Team clubhouse attendants and staff members were required to remain at their assigned location during games and movement between team clubhouse facilities was not permitted
Symptom screening and testing	Tier 1, asymptomatic: temperature and symptoms screened* at least twice per day, diagnostic testing^†^ every other day
	Tier 2, asymptomatic: home symptom screening, facility health screening upon entry to stadium or club facility, diagnostic testing at least two times per week
	Tier 3, asymptomatic: home symptom screening, facility health screening upon entry to stadium or club facility, no routine diagnostic testing
	Symptomatic or close contact of known COVID-19 case: clinical assessment, person is isolated, expedited diagnostic testing within 24 hours
Isolation of persons testing positive and quarantine of close contacts	Persons testing positive may be released from isolation provided the following criteria are met:
	• Two negative diagnostic test results, taken ≥24 hours apart
	• Afebrile ≥72 hours without the use of a fever suppressant and respiratory symptoms have improved (as documented by a clinician)
	• Completion of at least one antibody test after the positive diagnostic test result
	• Team medical staff members conclude person is no longer at risk for transmission
	• Local regulations are satisfied
	Close contacts may be released from quarantine provided the following criteria are met:
	• Negative diagnostic test results
	• Asymptomatic
	• Agreeing to participate in enhanced monitoring (e.g., more frequent temperature checks) by the team’s medical staff members for ≥10 days and daily diagnostic testing for 7 days after the exposure
Face masks	All persons must wear masks when in club facilities except when engaged in strenuous physical activity such as:
	• On the field, in the bullpen, or in the dugout
	• During games or practices
Social distancing	All players and staff members were required to maintain social distancing in club facilities
	Teams were advised to interact with one another only during gameplay, to avoid unnecessary physical interactions (e.g., high fives) and to avoid large group activities outside practices and games
	All players and staff members were encouraged to maintain social distancing outside of club facilities and to avoid activities that involved large groups or were primarily indoors
Environmental cleaning and disinfection	Routine cleaning of club facilities in accordance with CDC guidelines^§^
	Immediate cleaning and disinfection of club-controlled areas accessed by person with symptoms or diagnostic testing indicative of COVID-19

**Abbreviation:** COVID-19 = coronavirus disease 2019.

* Signs and symptoms screened included fever, shortness of breath or difficulty breathing, cough (new onset or worsening), headache, chills, sore or scratchy throat, new loss of taste or smell, muscle pain, nasal congestion, runny nose, nausea or vomiting, diarrhea, gastrointestinal distress or upset stomach, fatigue or weakness, swelling of the toes or lower extremities, chest tightness or pain, swollen lymph nodes or glands, abdominal pain, and rash or discolored and swollen toes (“COVID toes”).

^†^ Saliva or nasopharyngeal specimens were tested for SARS-CoV-2 using a reverse transcription–polymerase chain reaction test at the Sports Medicine Research and Testing Laboratory, Utah and the Rutgers Clinical Genomic Laboratory, New Jersey.

^§^ https://www.cdc.gov/coronavirus/2019-ncov/community/disinfecting-building-facility.html.

On day 1, team A traveled from location A to location C and played two games with team C (Figure).* On day 2, team A traveled from location C to location B for a three-game series with team B commencing on day 4. Before game play on day 4, the index team A player (an asymptomatic tier 1 risk group member who received every other day testing, per protocol) received a positive SARS-CoV-2 real-time reverse transcription–polymerase chain reaction (RT-PCR) test result from collection on day 2. The player was isolated at location B, and the Philadelphia Department of Public Health was notified, who led the outbreak investigation. After identification of the first confirmed COVID-19 patient on team A, all tier 1 and tier 2 players and staff members with known close contact with persons with COVID-19 on teams A and B were tested for SARS-CoV-2. Tier 2 staff members without known contact with a person with confirmed COVID-19, and staff members at facilities providing services for team A were offered voluntary diagnostic testing. Teams A, B, and C players and staff members received a diagnosis of outbreak-related COVID-19 if they had a positive SARS-CoV-2 RT-PCR test result during days 2–11 of the outbreak. Saliva specimens were collected by trained personnel. SARS-CoV-2 RT-PCR testing was conducted at the Sports Medicine Research and Testing Laboratory (Utah) or the Rutgers Clinical Genomic Laboratory (New Jersey). Translational Genomics Research Institute (Arizona) completed whole genome sequencing on residual diagnostic samples using standard methods (*3*). Sequence reads were aligned and compared with a Wuhan reference strain; all variants were identified and compared with the global GISAID SARS-CoV-2 database (>80,000 genomes). Investigators received from MLB a deidentified line list of team members with diagnosed COVID-19, a timeline of the outbreak response, the duration of on-field play by potentially infectious persons (within 24 hours before the date of collection of the test-positive specimen), contact tracing procedures, and the MLB health and safety protocols.

**FIGURE:**
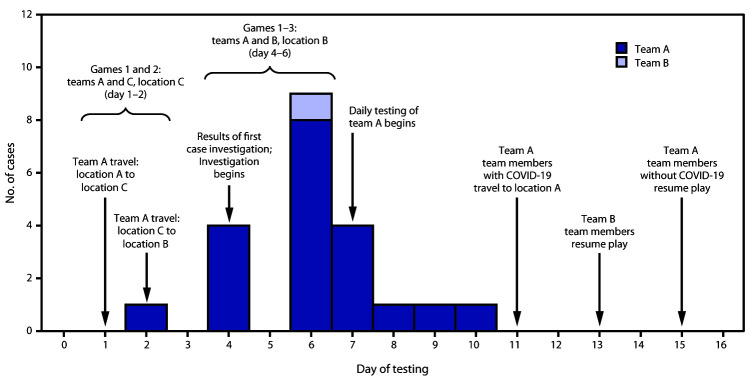
Dates of testing and events during a COVID-19 outbreak among professional baseball players and staff members (N = 21) — Major League Baseball, United States, 2020 **Abbreviation**: COVID-19 = coronavirus disease 2019.

After game 2 (day 5) of the three-game series at location B, three additional asymptomatic team A players received positive SARS-CoV-2 test results and were isolated. Immediately after game 3 (day 6), an additional eight team A players and staff members received positive test results, for a total of 12 team A players and staff members. MLB initiated daily testing for all team A and team B tier 1 and 2 employees for the outbreak duration. During days 7–11, eight more team A employees received positive test results. Overall, 20 COVID-19 cases were diagnosed among team A tier 1 players and staff members and one team B tier 2 employee; that employee’s official duties required indoor interaction with team A. Umpires and team C tier 1 employees continued with every other day testing and no further positive tests were recorded during the outbreak. Testing was available for non-MLB staff members(e.g., hotel staff members) who had possible interaction with team A. None of the 56 non-MLB staff members who were tested received positive SARS-CoV-2 test results.

Among 20 residual samples, 18 were suitable for whole genome sequencing. Among these, 17 had identical consensus sequences, and one showed a single nucleotide variant. These data were consistent with a single introduction resulting in a super-spreading event (*4*). Phylogenetic comparisons placed these 18 genomes in a large clade dominated by genomes sequenced from the southeastern United States.

In total, 146 MLB employees were exposed to SARS-CoV-2, including 68 associated with team A, 31 with team B, 38 with team C, and nine umpires; among these, 21 persons received positive SARS-CoV-2 test results. On average, testing results were available within 24 hours of collection (range = 12–48 hours). The overall attack rate was 14.4% (21 of 146), with team A, B, and C attack rates of 29.4% (20 of 68), 3.2% (one of 31), and 0% (zero of 38), respectively. Twenty of the 21 persons with diagnosed COVID-19 were tier 1 team A employees, and one was a tier 2 team B employee. Among the 19 of 21 persons with COVID-19 who were symptomatic, symptoms developed an average of 2.3 days after collection of the test-positive sample (range = 0–7 days). Potentially infectious persons had a total game time (e.g., bullpen, dugout, and on-field) of 40 hours 23 minutes and a total on-field play time of 11 hours 8 minutes at locations B and C; one on-field practice for team A occurred on day 3. Games scheduled with teams A and B were postponed on days 8–14. Team A persons with COVID-19 returned home on day 11 via private charter buses. Remaining team A employees traveled to location D on day 13. No additional cases were identified after day 11. Team A resumed play on day 15. Team B resumed practices on day 13 and play on day 14.

## Public Health Response

MLB isolated all players and staff members with COVID-19 at a separate location from those under quarantine. MLB coordinated with Philadelphia Department of Public Health to ensure rapid identification of cases and testing of contacts and to implement mitigation efforts. The lack of evidence for on-field transmission, as demonstrated by the absence of infections among opposing on-field team players and staff members, pointed to indoor exposures as the likely means of SARS-CoV-2 spread. Limitations identified in infection prevention practices led to MLB health and safety protocol revisions on day 16. Amendments included increasing cloth face mask use among players and staff members (i.e., at all times except on the field of play), limiting travel to essential staff members, and prohibiting visits to gatherings of large groups of persons (e.g., at bars and lounges).

## Discussion

COVID-19 outbreaks have been predominantly attributed to indoor settings with few investigations evaluating the spread of SARS-CoV-2 outdoors (*5*). Throughout five professional baseball games, asymptomatic, unknowingly infected players and coaches spent more than a cumulative 11 hours on the field. Although disease transmission were possible on the field, no opposing team players or coaches or umpires became ill during the outbreak. Interactions outside of on-field play were likely the source of spread, and SARS-CoV-2 transmission risk between baseball teams while on the playing field appeared low. Enforced health and safety protocols reduced interteam contacts and limited opposing team close contacts to brief interactions outdoors, potentially reducing the risk for transmission (*6*). Though one outbreak might not be representative of all scenarios faced by MLB, by mid October 2020, only 91 of 169,143 samples (0.05%) from 21 different teams returned positive test results; 57 (63%) of the 91 persons with positive test results have been players and 34 have been staff members. All patients recovered. No other COVID-19 outbreaks have spread to opposing MLB team members (*7*,*8*).

Social distancing policies likely limited the transmission of SARS-CoV-2 among staff members. Most persons with COVID-19 were tier 1 players and staff members, who had high likelihood of interpersonal contact in their roles within MLB. The one team B tier 2 employee who contracted COVID-19 closely interacted indoors with team A tier 1 employees who subsequently tested positive, likely increasing infection risk. Social distancing measures have been effective in a variety of settings (*9*), and this outbreak provides additional support for their use during sporting events, such as baseball games. When used, universal mask-wearing policies (*2*) provided additional protection for teammates. Consistent adherence to these policies off-field also might have contributed to protecting the communities hosting the games.

Mitigation strategies in the MLB health and safety protocols appear to have limited spread of COVID-19 beyond team A. However, even these multiple layers of protection are not infallible and containing the disease with isolation and quarantine is important in limiting the spread of COVID-19. The potential for presymptomatic transmission evidenced by MLB players and staff members reporting symptoms an average of 2.3 days after a positive test result highlights the importance of testing asymptomatic persons. The observed transmission within team A and evidence of a super-spreading event, led to updates in the health and safety protocols after the outbreak. Even with comprehensive COVID-19 mitigation efforts for sporting events, persons’ actions on and off the field are equally important in acquiring infection in the community and preventing transmission during games. This MLB outbreak investigation highlights the importance of employing multiple mitigation strategies to decrease risk for the person, the team, and the venue staff members.

Other professional sports leagues have adopted “bubble” strategies, (i.e., tightly controlled campus environments), to limit the exposures of players and staff members (*10*), whereas MLB and the players chose to play within their communities, enabling them to interact with persons outside of MLB including family members and the general public. Limited information on activities and exposures of players and staff members while off-field and in the community were available for this investigation. Some of the strategies employed, including frequent testing and dedicated contact tracing might not be realistic options for most nonprofessional teams. However, the multilayered strategy of mitigation measures used by MLB may have limited the spread to, within, and across teams.

Implementation of CDC COVID-19 mitigation strategies, particularly mask wearing and social distancing, as included in the MLB health and safety protocols, might provide nonprofessional baseball teams a means to play while reducing SARS-CoV-2 transmission between teams. However, in order to limit the spread of COVID-19 transmission both within and across teams, mitigation actions outside of game activities are also important. Willingness to adapt play based on knowledge of community transmission, and adherence to community mitigation strategies might allow sporting activities to resume. Persons engaged in similar sports might be able to implement some of these policies, e.g., within high schools and club teams, to provide a safer environment for all participants.

**Summary**

**What is already known about this topic?**

Mitigation strategies decrease the spread of communicable diseases. Data on their effectiveness to prevent and mitigate SARS-CoV-2 transmission during outdoor sporting events are limited.

**What is added by this report?**

During the 2020 season, Major League Baseball instituted a multilayered COVID-19 prevention and mitigation strategy. In an outbreak among 20 baseball players and staff members on a single team, no secondary transmission during field play between two opposing teams occurred. Interactions outside of game play were the likely source of transmission within the team.

**What are the implications for public health practice?**

Adherence to COVID-19 mitigation measures on and off the field has important implications for infection prevention in comparable sports teams, including professional, amateur collegiate, high school, and club baseball and softball teams.

## Footnotes

* The Figure shows the day of testing, whereas the results presented in the text provide the day of receipt of a positive test result.
